# The Development and Characterisation of ssDNA Aptamers via a Modified Cell-SELEX Methodology for the Detection of Human Red Blood Cells

**DOI:** 10.3390/ijms25031814

**Published:** 2024-02-02

**Authors:** Hayley Costanzo, James Gooch, Sireethorn Tungsirisurp, Nunzianda Frascione

**Affiliations:** Department of Analytical, Environmental & Forensic Sciences, Faculty of Life Sciences & Medicine, King’s College London, London SE1 9NH, UK; hayley.costanzo@kcl.ac.uk (H.C.); james.gooch@kcl.ac.uk (J.G.); sireethorn.1.tungsirisurp@kcl.ac.uk (S.T.)

**Keywords:** aptamer, forensic, blood, Cell-SELEX, massively parallel sequencing, binding affinity

## Abstract

Blood is one of the most commonly found biological fluids at crime scenes, with the detection and identification of blood holding a high degree of evidential value. It can provide not only information about the nature of the crime but can also lead to identification via DNA profiling. Presumptive tests for blood are usually sensitive but not specific, so small amounts of the substrate can be detected, but false-positive results are often encountered, which can be misleading. Novel methods for the detection of red blood cells based on aptamer–target interactions may be able to overcome these issues. Aptamers are single-stranded DNA or RNA sequences capable of undergoing selective antigen association due to three-dimensional structure formation. The use of aptamers as a target-specific moiety poses several advantages and has the potential to replace antibodies within immunoassays. Aptamers are cheaper to produce, display no batch-to-batch variation and can allow for a wide range of chemical modifications. They can help limit cross-reactivity, which is a hindrance to current forensic testing methods. Within this study, a modified Systematic Evolution of Ligands by Exponential Enrichment (SELEX) process was used to generate aptamers against whole red blood cells. Obtained aptamer pools were analysed via massively parallel sequencing to identify viable sequences that demonstrate a high affinity for the target. Using bioinformatics platforms, aptamer candidates were identified via their enrichment profiles. Binding characterisation was also conducted on two selected aptamer candidates via fluorescent microscopy and qPCR to visualise and quantify aptamer binding. The potential for these aptamers is broad as they can be utilised within a range of bioassays for not only forensic applications but also other analytical science and medical applications. Potential future work includes the incorporation of developed aptamers into a biosensing platform that can be used at crime scenes for the real-time detection of human blood.

## 1. Introduction

Within a forensic setting, the term ‘body fluid’ is used to describe suspected stains that are recovered from a surface outside of the originating body and often form the key evidence recovered from crime scene investigations [1]. Biological fluids, such as blood, saliva and semen, are frequently found at crime scenes and are an important type of trace evidence in casework. The detection of biological fluids can hold a high degree of evidential value, providing information about the nature of the crime and can lead to personal identification [2]. Blood is one of the most commonly found biological fluids at crime scenes and it is regarded as the fluid of highest evidential value [3]. For example, the presence of blood at a scene can indicate that the crime may have been violent in nature and could provide a source of genetic material that can aid in the identification of either an offender or a victim.

When evaluating the presence of blood at a crime scene, two factors must be considered: localisation and identification. Initial localisation ensures that a stain is found, with the subsequent identification confirming the nature of the stain, for example, a biological fluid origin or a non-biological stain that is of less forensic interest. Localising a blood stain is crucial to a case as stains can be missed during manual search due to small volume or indistinguishability against certain backgrounds [4]. Utilising a robust and reliable testing method for the localisation of blood is paramount for detecting potential evidentially significant material [5]. Without such tests, crime scene searches may result in missed evidence, which can seriously compromise cases, leading to miscarriages of justice [6,7]. The location and distribution of blood staining can provide additional relevant investigative information. The features of a stain can indicate the actions and movements of people or objects during the course of a crime, for example, where in a room a crime was committed and blood was shed, or a trail of blood leading to an additional crime scene. This links in with the morphology of a stain, which can also provide crucial evidence, such as blood spatter patterns and footwear marks, which can supply law enforcement and forensic practitioners alike with information regarding movement throughout a crime scene and where participating people may have been located during the time of bodily fluid deposit to the scene [8]. The current method for body fluid identification at crime scenes begins with a visual examination, with the naked eye or with Alternative Light Sources (ALSs), whereas examination in the laboratory consists mainly of visual searching with optical microscopy [9]. ALSs have also been employed within the field as a means for identifying latent stains, as they allow stains to be visualised that would otherwise go unseen, due to the low concentration or because they are present on a dark background [4]. However, ALSs are not specific to body fluids and can cause other non-body fluid stains to fluoresce that are present on the surface, such as hand cream, at a similar wavelength to semen, making the two indistinguishable [10]. 

Once a stain has been localised, attribution is necessary to guide further testing and sample analysis. Stains that are either visible to the naked eye or found via ALSs are then subject to presumptive testing. A presumptive test can be defined as a screening test carried out to detect a specific substrate, in this case blood, that is commercially available for use at crime scenes or within a laboratory environment [11]. Commercially available testing methods for the detection and attribution of blood, such as the Kastle–Meyer test, are often used at crime scenes. These tests use simple biochemical reactions to generate a colourimetric change in the presence of blood, allowing for a rapid indication of the substrate [12]. Presumptive tests for blood often utilise the oxidation of haem to catalyse substrate-specific reactions that generate a colour change [13]. Presumptively testing stains provides rapid intelligence on the nature of the offence and can guide an investigation early on. They also highlight areas of staining that may contain a valuable source of genetic material that can be used for downstream genetic profiling to associate or exonerate a suspect. Furthermore, attribution of the stain early on can help to direct the investigative strategy in the laboratory by a forensic analyst and can save time and resources if a stain has already been confirmed as a specific bodily fluid. 

Presumptive tests are usually sensitive but not specific, so small amounts of the substance can be detected. However, false-positive results are often encountered during casework and can be misleading when observed. Alongside the lack of specificity, the major drawback of presumptive tests, such as the Kastle–Meyer test, is that they have been reported as being DNA destructive, which could affect downstream profiling, rendering the evidence far less significant than if a DNA profile is generated from the stain [13,14]. If a positive result is obtained from a presumptive test, then confirmatory tests are carried out, which are generally more specific and reliable. A confirmatory test can be used to confirm the presence of blood, but these tests are often deemed too expensive for routine casework, so exhibits that test positively during a presumptive test are often sent directly for DNA profiling [15]. Currently employed presumptive and confirmatory tests present a lack of specificity and the ability for multiplexed analysis, hence the need for a novel detection and attribution method that can give localisation and stain attribution in real time. Aptamers have gained attention in recent years for their suitability towards analytical science as they have the capability to bind not only nucleic acid targets but a variety of targets, from small molecules to whole cells [16]. Aptamers are single-stranded DNA or RNA sequences capable of undergoing selective antigen association due to their three-dimensional structure formation [17]. Aptamers are analogous to antibodies in terms of target binding affinity but possess features that could allow them to become a preferential choice over antibodies [18,19]. Aptamers are mass-produced with ease using automated solid-phase synthesis techniques, which produces highly purified oligonucleotides in only a few hours and at a lower cost than antibody generation methods [17,20]. Furthermore, aptamers have greater thermal stability, resulting in a longer shelf-life without losing activity and can also be easily transported and stored [21]. 

Within this study, aptamers were developed via a modified Cell Specific Systematic Evolution of Ligands by Exponential Enrichment (Cell-SELEX) protocol previously outlined by Sefah et al. [22]. Modifications were made to the protocol to optimise the method for use with isolated red blood cells (RBCs). This entailed adding the target cells to a random DNA library and allowing the target to bind to the sequences, followed by the removal of the non-binding strands via wash steps, then amplification of the binders via polymerase chain reaction. The amplification step increases competition for the target between remaining binding strands, so only the aptamers with the highest affinity remain bound to the target. This was performed in a series of rounds to ensure that the only sequences remaining within the pool were of the highest affinity and specificity for the target. Throughout these rounds, the molar ratio between the target and library was altered in order to adjust selection stringency to enrich the high-affinity aptamers. Finally, massively parallel sequencing (MPS) was used to sequence the binding pools and identify the most suitable aptamers. MPS was used to analyse the enriched aptamer pools due to the in-depth sequencing information obtained and the lack of time-consuming Sanger sequencing. Using two bioinformatic programmes, Galaxy and AptaSUITE, the MPS datasets were processed to elucidate binding sequences [23,24]. The Galaxy pipeline used within this project was developed in house and was adapted for use with the specific library for this SELEX selection [25]. Aptamer binding capability and selectivity were investigated using Quantitative Polymerase Chain Reaction (qPCR) and fluorescent microscopy. Aptamers with high binding capability have the potential to revolutionise in situ screening as they could allow for a higher specificity and sensitivity of detection for blood compared with the currently used methods, whilst remaining a cost-viable option compared to immunoassays that are commercially available. 

## 2. Results and Discussion

### 2.1. Cell-SELEX Aptamer Selection 

**Scheme 1:**
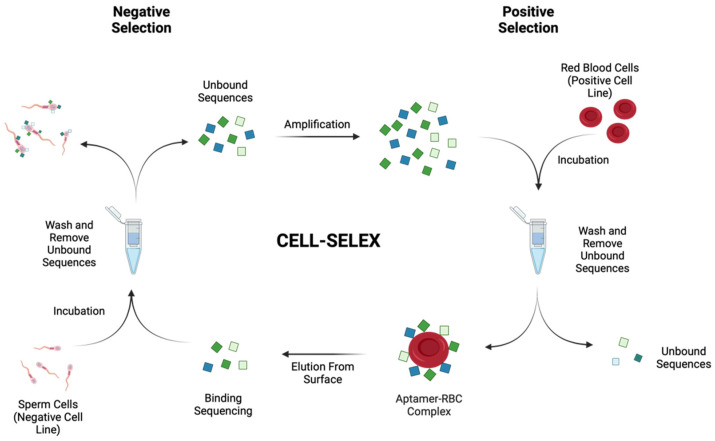
Cell-SELEX with negative selection. A schematic representation of the Cell-SELEX process used to generate high-affinity aptamers against human red blood cells. Negative selection rounds were conducted from round 7+, using isolated human sperm cells as a control cell line. Green and blue squares represent the randomised oligonucleotide library.

The Cell-SELEX reported within this work is a modified version of the systematic evolution of ligands by an exponential enrichment protocol previously reported by Sefah et al. [22] (Scheme 1). This protocol was initially modified in order to accommodate the use of isolated RBCs as the target cell. First, the isolation of RBCs from whole blood via a cell washing based protocol was used opposed to the cell culture methods originally outlined within the protocol [26]. Secondly, cell counting was conducted using a Countess II automated cell counter, which also confirmed the viability of cells to be >95%. Successful isolation and removal of other whole blood components were confirmed via microscopic examination. This was a critical step, as the ideal aptamer candidates would be specific for red blood cells only, opposed to components of blood that could also be found within other biological fluids, such as enzymes or proteins.

In order to better enrich the binding pool throughout subsequent rounds of selection, stringency measures were employed. The five main variables explored were donor rotation, RBC count, incubation time, wash steps and, finally, negative selection (Table 1). To ensure a representative population was used, donors of different blood-grouping types, biological sex and geographical ancestry were selected. Opposed to pooling RBCs from five donors for use within each round, a single donor per round was used, and rotation between the five donors over the ten rounds was carried out. This was carried out in order to remove possible binding sequences that bound to markers on the red blood cells that may not be present within the entire population and instead only capture sequences with the capability to bind to RBCs from multiples donors. It is reported that the red blood cell population itself is constantly in flux but can also vary between individuals [27]. When considering the forensic application of generated aptamers, it is vital that the selected candidates are able to detect blood in a confirmative manner; hence, they must be able to bind to red blood cells of a representative population. Secondly, the RBC count was lowered throughout the rounds of SELEX, hence increasing competition for binding, ensuring that only candidates with the highest affinity for the target remain in the selection pools. As suggested in the original Cell-SELEX protocol [22], cell count reduction is a useful tool in order to enrich high-affinity candidates within the pool by increasing competition for binding regions of the target. Therefore, the initial selection cycle started with 1 × 10^7^ cells and was decreased until the seventh round of selection. From then on, the number of cells was kept at 1 × 10^6^ cells whilst other parameters were changed. A single cubic millimetre of blood can contain between 4 and 6 × 10^6^ red blood cells; therefore, a starting cell concentration of 1 × 10^7^ cells was chosen in round 1 of selection. This was to ensure that there would be a high enough concentration of RBCs to capture initial binding sequences in a small volume of blood [28]. Throughout subsequent rounds, this number was then reduced to 1 × 10^6^ cells to increase competition for binding and ensure that the selected aptamers would be of the highest affinity binding to the cells. This is not only important for a forensic application, where small volumes of blood are often detected, but also in medical applications where point-of-care testing utilises a low-volume sample input, such as a finger prick sample. 

After library incubation, the elution stage of SELEX typically involved three separate washes in order to remove unbound sequences from the aptamer–target complexes. Within this work, the number of washes was kept constant (×3) until round 8, where the number of washes was increased by one wash from round 8 to 10. The increase in washes was chosen in latter rounds of selection to add a further layer of stringency. This should aid the physical removal of lower-affinity sequences that may still be present within the binding pool and allow for a greater level of binding (and, hence, enrichment) of higher-affinity candidates. 

The modified Cell-SELEX protocol also involved the incorporation of four negative selection rounds from round 7 to 10. The negative cell line chosen was isolated human sperm cells. When considering the application of generated aptamers for the forensic detection of blood, it is of the utmost importance to avoid cross-reactivity with sperm. Within forensic casework, mixed biological fluids are often encountered within evidence recovered and can pose a difficulty for forensic examiners [29]. For example, in cases of sexual assault, blood and semen are often found within a complex mixture or in similar locations [30]. Therefore, by using sperm cells as the negative control line, aptamer candidates that show a level of binding to sperm cells would be removed from the pool, hence allowing only RBC-specific candidates to enrich. The number of isolated sperm cells used per round was kept the same as the number of RBCs. 

Within each selection round, a trial PCR was conducted in order to determine the optimum number of cycles for preparative PCR amplification. Once PCR amplification was conducted, strand separation was used in order to create ssDNA products, ready for incubation within subsequent rounds of selection. The number of PCR cycles chosen for the original round 1 library was 10 cycles, which corresponded to the clear, bright band, with no non-specific amplification occurring shown on an agarose gel. Throughout subsequent rounds of selection (when stringency measures were increased, therefore increasing competition within the pool and reducing sequence variety within the pool), a higher number of cycles was needed (16–18 cycles), indicating that from the first original selection, the amount of ssDNA decreased, therefore requiring a higher amplification cycle number. 

Strand separation was achieved through the use of Pierce NeutrAvidin agarose resin that has the capability of capturing the biotinylated antisense strand of the dsDNA amplification products. Through a washing procedure and the addition of NaOH, the fluorescently labelled sense ssDNA strand could be collected and quantified prior to the subsequent rounds of SELEX. All rounds produced a sufficient amount of ssDNA to proceed to the next round (400 μL of 1 μM ssDNA pool), giving an indication that PCR amplification was successful. After the first cycle of Cell-SELEX, a concentration of 2367.31 nM ssDNA was obtained. This high concentration was likely due to the lack of stringency measures employed within round 1, meaning high numbers of aptamers were bound to the RBCs. Within the second round of selection, the RBC count was halved from 1 × 10^7^ to 5 × 10^6^ cells and, as a result, the amount of ssDNA recovered was almost halved as the stringency was increased. Recovered ssDNA from round 7, where negative selection was first introduced, was double that recovered in round 6, indicating a greater amount of ssDNA captured. This could be due to the fact that negative selection removed a greater number of non-specific cell binding sequences, therefore allowing RBC-specific candidates to bind and enrich within the pool. From round 7+, the amount of ssDNA recovered was again reduced, giving an indication that the stringency was eradicating a large proportion of aptamers from the pool, therefore leaving only those with a high affinity for the target. 

### 2.2. Massively Parallel Sequencing and Data Processing 

In order to elucidate possible binding sequences within the pools and monitor the enrichment of the library throughout the rounds, MPS was used. MPS was chosen for use within this study in order to obtain in-depth sequencing knowledge regarding the pools and to avoid the use of Sanger sequencing, which can be laborious [31]. Already being employed within forensic laboratories, MPS boasts the advantages of being able to simultaneously analyse large pools, as well as being a robust method that requires relatively simple sample preparation [32,33]. Rounds 6, 8 and 10 were chosen for high-throughput sequencing via this method. Round 6 was selected as this round was the first round, whereby the number of RBCs was kept consistent at 1 × 10^6^ cells and the last round prior to the incorporation of negative selection within the rounds. Therefore, by round 6, there should be an enrichment of an aptamer population, but subsequent rounds increased in stringency measures, meaning further enrichment of binding candidates would be seen. Rounds 8 and 10 were chosen for sequencing to firstly see the effect of incorporating negative cell line selection and secondly to determine if any candidates had enriched during the latter stages of the selection process. 

**Figure 1:**
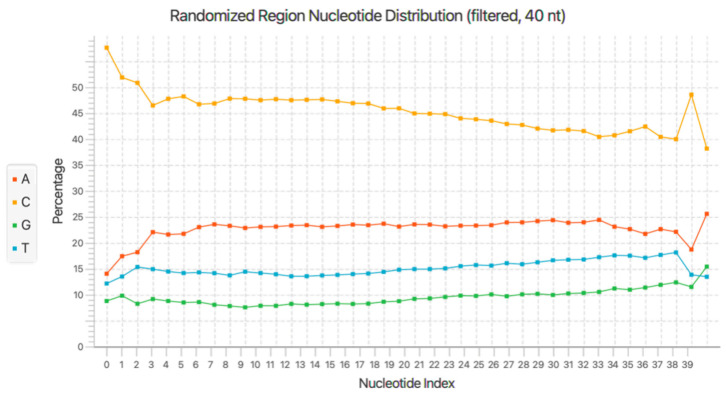
Binding pool base distribution. Nucleotide distribution of the randomised region of the round 10 binding pool generated through Cell-SELEX. Results obtained from AptaSUITE [24].

In order to elucidate the 76 bp aptamer sequences within the MPS data, significant data manipulation was required. Any regions of DNA associated with the MPS library preparation, such as the addition of indexes and any sequencing artefacts that may be present within the dataset, were removed. Firstly, Galaxy was used with an automated, custom Cell-SELEX pipeline that is capable of processing FASTQ files (both forward and reverse read files) that were generated as a result of MPS. Stepwise information for this custom pipeline has been reported in 2021 by Gooch et al. [25]. In order to validate results obtained from the Galaxy workflow, AptaSUITE was employed in order to gain further insight into features of the pools, such as base distribution and enrichment. Figure 1 shows the randomised region (40 base) nucleotide distribution by base. This shows that an average of 46% of the pool comprised cytosine, which indicates that the target site on the RBC has a strong interaction with cytosine. Despite showing a lower percentage of guanine in the pool, Section 2.3 below outlines the predicted G-quadruplex-forming regions within the enriched candidates. 

Processing of the data showed that a total of ~200,000 reads were achieved per sample during sequencing for each round processed (6, 8 and 10). Within the round 10 binding pool, a total of 3365 sequences were found to be replicated multiple times in the dataset, with 9 sequences showing enrichment from the round 6 pool (Table 2). Due to their enrichment indicating a greater level of binding to red blood cells, these nine candidates were selected as the most promising aptamer candidates from the selection process.

Whilst N1 and N4 both demonstrated enrichment profiles from round 6 to 10, N2 and N3 do not display a full enrichment profile. This lack of full profile is attributed to the read count during MPS, which may only capture a proportion of the full sample. When considering that the copy number is likely to still be low during round 6 or 8 due to lack of enrichment, it is, therefore, most probable that the sequence has been omitted during MPS due to the ~200,000 reads being conducted per sample. In light of this lack of enrichment profile for N2 and N3, it was decided that N1 and N4 would be investigated further. Both sequences contain the two 18-base primer flanking regions at both the 5′ and 3′. Despite contradicting literature on whether or not these regions should be included in the final sequence (opposed to just the 40-base random region), it was decided that, given these regions could have been involved in original binding to the target, they should be included as part of the total sequence for subsequent analysis. These two aptamers were, therefore, selected for further investigation due to the confirmed enrichment profiles demonstrated during selection. 

### 2.3. Aptamer Structure Analysis

#### 2.3.1. Secondary Structure Predictions

**Figure 2:**
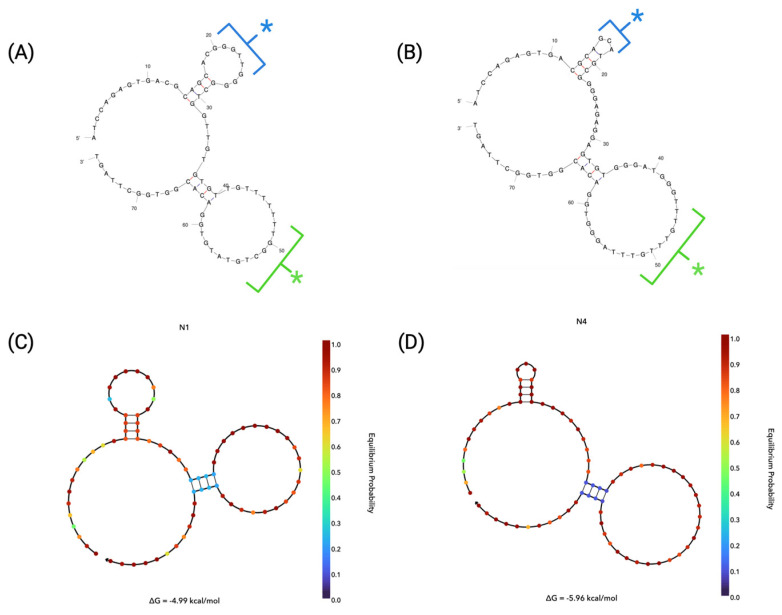
Secondary structure predictions. Predicted secondary structures of aptamers using Mfold software of (**A**) N1 and (**B**) N4 [35]. On each aptamer structure, the 5′ loop structure is indicated by a blue asterisk and the 3′ loop structure is indicated by a green asterisk. NUPACK equilibrium probabilities plots using NUPACK software are shown for (**C**) N1 and (**D**) N4 [36].

The secondary structure of an aptamer plays a vital role in the binding of the sequence to its target ligand. It is reported that the binding strength of an aptamer can be attributed to its structure, such as being in a hairpin structure, G-quadruplexes or T-junctions [34]. In order to better characterise N1 and N4, a computer-based prediction software, Mfold, was used to predict the secondary structure of the aptamers based on the minimum free energy [35]. A secondary prediction software, NUPACK (Cloud Alpha), was then utilised in order to predict the secondary structures of N1 and N4 as well as to predict the probabilities of the formation of the structures obtained [36]. Structure predictions were estimated using a folding temperature of 25 °C with 0.137 M Na^+^ and 0.005 M Mg^2+^ in order to replicate original binding conditions during selection during all simulations. The secondary structures chosen were those that displayed the lowest free energy of formation (Gibbs Free Energy, ΔG) and, therefore, the most probable secondary structures in their unbound state. Structures were also assessed on the similarity of structure prediction between Mfold and NUPACK. The resulting secondary structures obtained from Mfold showed that N1 (Figure 2A) had an estimated ΔG = −4.99 kcal/mol, whilst N4 (Figure 2B) had an estimated ΔG = −5.96 kcal/mol. The structures with the lower ΔG values were, therefore, considered for each of the presented aptamer candidates. This is due to the assumption that the most spontaneous and stable structure will form when lower dissipated energy is observed [37]. 

Both N1 and N4 display similar typical hairpin structures, comprising stems and two loops each. Both aptamers display two regions of internal nucleotide pairing, which contributes to the formation of both the major and minor loops of the N1 and N4 aptamers. Whilst the structures are similar, they display minor differences in both of their 5′ loops and 3′ loops (Figure 2A,B). The similarity in structures between the two aptamers may give an indication that both aptamers have the ability bind to the same, or similar, targets on the surface of the red blood cells [38]. 

When considering the equilibrium probability profiles of N1 and N4 (Figure 2C,D, respectively), it is shown that both structures display a similar probability of formation for the structure throughout, with the only exception being in the 3′ loop stem formation for both aptamers. Here, it can be seen that the stem of the 3′ hairpins shows lower probability of formation, as indicated by the blue-coloured base representations at those positions. Therefore, this indicates that the 3′ loops of both N1 and N4 may not be as stable structures within the aptamer under the given conditions as the rest of the sequence. The probability of loop formation has also been predicted by Mfold, as shown in Figure 2A,B, with the probability of individual nucleotides to participate in base pairs being shown via the colour of the bond. A red bond denotes a ~0.999 probability and a blue bond denotes 0.500 probability of base pairing [35].

#### 2.3.2. GQRS Mapper

QGRS Mapper was used to identify G-quadruplex-forming regions within the aptamer sequences of N1 and N4 (Table 3) [39]. QGRS Mapper is a web-based server that can predict quadruplex-forming G-rich sequences within nucleotide sequences. G-quadruplex structures are non-canonical oligonucleotide structures that are stabilised via stacking interactions of G-quartets, in which four guanines are assembled in a planar arrangement [19]. Their structure can vary widely, but they can be formed by either one, two or four strands of DNA, with the direction of strands varying (such as parallel or antiparallel) [40]. They are of great interest within aptamer development generally, as the presence of G-quadruplex structures within a sequence can indicate a greater thermal and chemical stability compared with an unstructured sequence, making it a more suitable candidate for inclusion within an analytical probe. Table 3 shows the position on the sequence, length of predicted G-quadruplex, sequence and G-score determined by Mfold. The G-score assigned to each quadruplex-forming guanine-rich sequence is a scoring system that evaluates the likelihood of a QGRS forming a stable G-quadruplex. Therefore, higher-scoring sequences will make better candidates for G-quadruplexes. 

Both N1 and N4 display two G-quadruplex-forming regions, which can give an indication of stability and aid with subsequent 3D structure predictions. It has also been suggested that the presence of these regions within N1 and N4 can also aid with electrostatic interactions of the aptamer to positively charged binding ligands. This is due to the fact that the structures will have a 2-fold negatively charged density per unit length compared with the duplex DNA [19]. This can assist with a better understanding of the aptamer–target interaction and with optimising the binding event. 

#### 2.3.3. Circular Dichroism 

**Figure 3:**
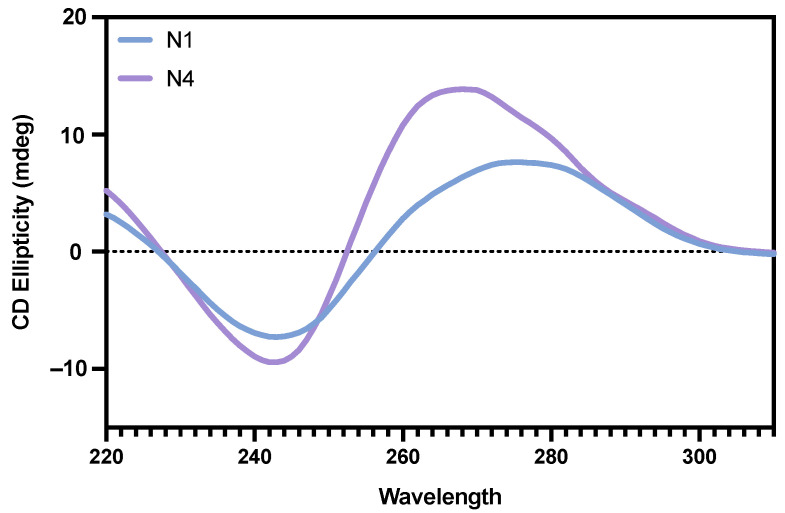
Circular dichroism spectra. CD spectra of both N1 (blue) and N4 (purple) aptamers in their folded, unbound state.

Circular Dichroism (CD) is a spectroscopic tool that can be utilised for obtaining structural information of nucleic acids through their characteristic spectra [41]. CD is a highly useful tool as it can provide reliable information about the structure of DNA and can characterise G-quadruplex structures [42]. Therefore, CD analysis was performed for both N1 and N4 in order to further study the secondary structure of each aptamer. For the N1 aptamer, the spectra show an ellipticity minima at ~240 nm and a maxima at ~280 nm (Figure 3). This spectrum is indicative of a B-DNA helix structure [43]. The N4 aptamer shows an ellipticity minima at ~240 nm and a maxima at ~265 nm, which is indicative of a parallel quadruplex structure [44]. 

### 2.4. Binding Characterisation

#### 2.4.1. Fluorescent Microscopy

**Figure 4:**
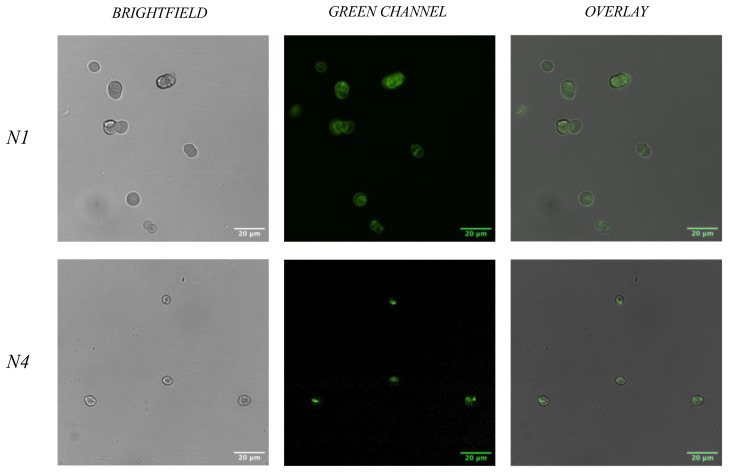
Fluorescent microscopy images with isolated RBC. Fluorescent microscopy analysis of aptamers N1 and N4 with red blood cells. Cells were labelled with 20 μM 6-FAM labelled aptamer. The fluorescent overlay shows areas of aptamer labelling of both N1 and N4 on the RBC surface. Slides were visualised under ×40 objective lens. Scale bar = 20 μm.

In order to confirm the binding of developed aptamer sequences to their target—RBCs—a fluorescent bioassay was performed. To ensure that the aptamer sequences are able to bind to multiple donors (representative of a wider population), a total of three healthy donors were used for this assay, with whole blood being pooled prior to the isolation of RBCs. During this assay, isolated human RBCs from the pooled donors were incubated with 20 μM of 6-FAM labelled N1 and N4 aptamers, independently. When considering wider application of RBC binding aptamers, it is important to recognise the need for multiple donors, to ensure that the aptamers can bind to RBCs that may demonstrate a different surface proteome, therefore, ensuring that they are ‘universal’ binders. As shown in Figure 4, both aptamers show a successful level of binding to the target, as shown through the green fluorescent signal seen over the RBCs. When overlaying the images obtained from the RBCs using only the brightfield with the images obtained from the green channel excitation filter, it appears that the fluorescence emission from the 6-FAM aptamers is at the RBC surface, indicative of a binding event. In order to achieve visible labelling of the cells, a concentration range of each aptamer was first tested from 5 nM to 20 μM. It was concluded that using a concentration of 20 μM was optimal in order to obtain clear fluorescent labelling for both aptamers. When comparing N1 and N4 labelling of the RBCs, N1 showed a greater level of labelling than N4. This suggests that the target binding site of the N1 aptamer could be more prevalent on the RBC surface than that of the N4 aptamer, therefore suggesting a greater affinity of the N1 aptamer for the RBC. Despite this difference, both aptamers displayed a level of binding to RBCs, showing their applicability for use within a forensic setting, where rapid, optical detection is desired. 

**Figure 5:**
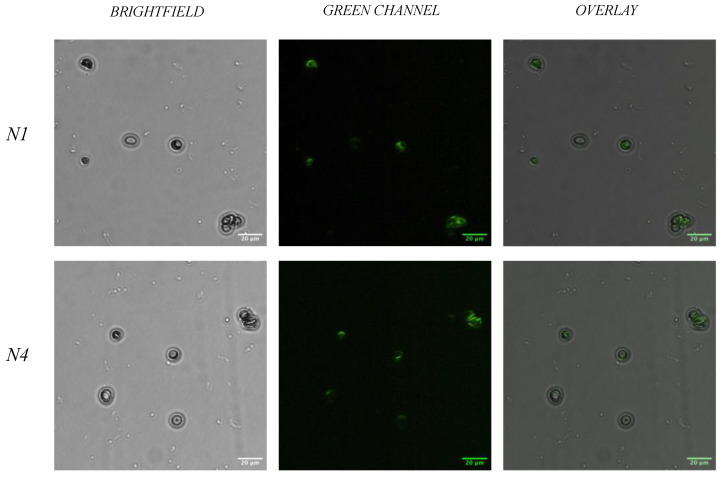
Fluorescent microscopy images with whole blood. Fluorescent microscopy analysis of aptamers N1 and N4 incubated with whole blood. Whole blood was incubated with 20 μM 6-FAM labelled aptamer. The fluorescent overlay shows areas of aptamer labelling of both N1 and N4 on the RBC surface. Slides were visualised under ×40 objective lens. Scale bar = 20 μm.

When considering the use of N1 and N4 in a forensic setting, it is vital to assess the binding capabilities of the aptamers within whole human blood. Therefore, a second fluorescent bioassay was performed as above, however, utilising whole blood opposed to the isolated RBCs. Briefly, each 6-FAM labelled aptamer solution (at 20 μM) was added to whole blood and left to incubate for 15 min. Post incubation, the solution was centrifuged to remove unbound sequences, and washed (×3) prior to visualisation on a slide. As demonstrated in Figure 5, both N1 and N4 retain the ability to bind to red blood cells when in the presence of human serum and other components of whole human blood during incubation. Whilst a minor decrease in the fluorescent signal can be observed on the surface of the RBCs when compared to incubation with RBC alone, the ability to detect a signal is sufficient to demonstrate the binding capabilities of both N1 and N4 in whole blood. The reported time for aptamer degradation by human nucleases varies depending on the structure and concentration of the aptamer [45,46]. More tightly folded aptamer structures will display a greater resistance to degradation due to limited solvent exposure, with shorter aptamers also displaying a quicker degradation [45,47]. When considering the application of these aptamers within a biosensor for the real-time detection of human blood, a signal output should be generated within minutes; thus, the level of binding seen in Figure 5 is deemed acceptable given that a signal was still produced after an incubation time of 15 min. A range of aptamers designed for various therapeutic targets have demonstrated their ability to bind to their targets and, thus, their stability within human serum [48,49,50]. Future optimisation of the N1 and N4 aptamers could look at modifications to protect them from nuclease hydrolysis, through methods such as linker modification or sugar backbone modifications [51,52]. Prior to the incorporation of these aptamers within a biosensor for use within a forensic workflow, additional validation studies would be completed to further assess stability and performance under various environmental and biological conditions. 

#### 2.4.2. Quantitative Polymerase Chain Reaction (qPCR)

**Figure 6:**
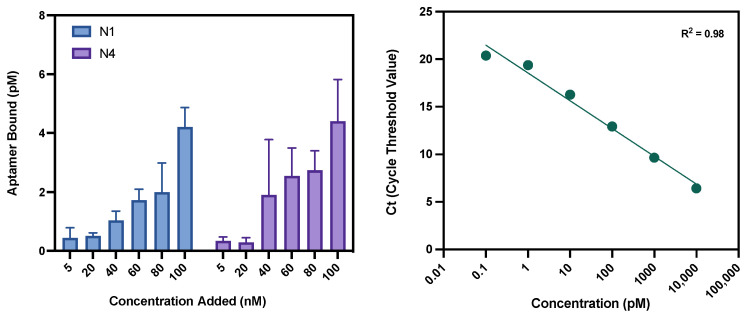
qPCR assay results and standard curve. **Left**: Binding characterisation of N1 and N4 via qPCR. Both aptamers display dose-dependent binding to RBCs, with a greater amount of aptamer bound when incubated with an increased concentration of each respective aptamer. **Right**: Standard curve obtained using qPCR. The average threshold cycle (Ct) is shown of three independent replicates for the template concentration range 10 nM–0.1 pM. Error bars = s.d., n = 3.

By using qPCR as a binding assay, the binding of an aptamer to a target can be quantified. qPCR is a sensitive technique that has been considered to be the ‘gold standard’ for the quantitative analysis of nucleic acids and has had successful use within aptamer binding characterisation studies to monitor dose-dependent binding [53,54,55,56]. In order to utilise this assay for N1 and N4, a range of concentrations of each aptamer was prepared: 100 nM, 80 nM, 60 nM, 40 nM, 20 nM and 5 nM. All reactions were conducted in triplicate. After incubation with human RBCs, the bound aptamers were cleaved from the surface and quantified using qPCR. Utilising the obtained average threshold cycles (Ct) from the aptamer control samples, a standard curve was established (Figure 6). To validate the standard curve, a positive control was used of a 50 pM solution of aptamer. This then correctly calculated the aptamer concentration from the obtained Ct generated, validating the method for use with N1 and N4. Both N1 and N4 display dose-dependent binding to RBCs, as demonstrated in Figure 6. Both aptamers showed the general trend of an increased concentration of aptamer bound to the RBCs as the concentration of aptamer added increased. A total of three healthy donors were used for this bioassay, with blood donation being pooled prior to RBC isolation. This ensures that a variety of RBC variations are present within the pool, capturing a range of possible surface proteins and, hence, possible binding sites.

Due to variation in protein expression on the RBC surface, the exact number of N1 and N4 binding sites is unknown and may vary between donors. Therefore, within this assay, a point of saturation is not reached for either N1 or N4 during these binding events. This has been attributed to the fact that using a whole cell as a target means that the number of binding sites is unknown and likely very high, meaning saturation of all binding sites is unlikely to occur, and the saturation point may vary amongst individuals. Secondly, depending on the binding site itself and the number per cell, full saturation may never be achieved if target sites become blocked due to steric hinderance of bound aptamers. However, being able to demonstrate dose-dependent binding validates the function of these aptamers as being able to bind to human RBCs. 

## 3. Materials and Methods

### 3.1. Reagents

For the isolation of RBCs from whole human blood, a cell wash buffer was prepared (21 mM TRIS, 4.7 mM KCl, 140.5 mM NaCl, 2 mM CaCl_2_, 1.2 mM MgSO_4_, 5.5 mM glucose, 0.5% Bovine Serum Albumin in sterile-distilled water (SDW)). The solution was then adjusted to pH 7.4. Additionally, 3.2% sodium citrate coagulation preservative was obtained from a BD vacutainer Plus tube (Oxford, UK). Safety lancets (26 G needle, Penetration Depth 1.8 mm) were obtained from VWR International (Lutterworth, UK). 

For the isolation of sperm cells from human seminal fluid, Percoll was obtained from Sigma-Aldrich (Dorset, UK). Human tubal fluid (HTF) was prepared as a ×10 solution via the addition of 29.655 g NaCl, 1.75 g KCl, 250 mg MgSO_4_·7H_2_O, 250 mg KH_2_PO_4_, 2.5 g glucose, 2.6 g HEPES, 1 g NaHCO_3_, 1.5 g CaCl_2_·2H_2_O and 3 g human serum albumin (HSA) to 350 mL SDW. The solution was adjusted to pH 7.4 and made up to 500 mL using SDW. Dilution of this concentrate in SDW was used to prepare all ×1 HTF solutions. 

For cell counting, Countess™ Cell Counting Chamber Slides (with Trypan Blue Stain) were obtained from Thermo Fisher Scientific (Waltham, MA, USA). Saline solution 0.9% was obtained from Severn Biotech Ltd. (Kidderminster, UK). 

For Cell-SELEX incubation and elution of sequences, a single-stranded DNA (ssDNA) library containing two fixed primer binding sites of 18 nucleotides in length and a randomised N40 base sequence region (5′-ATCCAGAGTGACGCAGCA-N40-TGGACACGGTGGCTTAGT-3′, HPLC purified) was obtained from Integrated DNA technologies (Coralville, IA, USA). Aptamer binding buffer was prepared through the addition of 4.5 g glucose, 1 g BSA, 100 mg tRNA and 5 mL of 1 M MgCl_2_ to 1 L of DPBS. 

PCR reagents, including ×10 PCR buffer, 10 mM dNTP mixture, 50 mM MgCl_2_ solution and Platinum Taq DNA polymerase, were obtained from Thermo Fisher Scientific (Waltham, MA, USA). Fluorescently labelled forward and biotin-modified reverse (Forward: 5′-6-FAM-ATCCAGAGTGACGCAGCA-3′. Reverse: 5′-biotin-ACTAAGCCACCGTGTCCA-3′, HPLC purified) primers were both obtained from Integrated DNA technologies (Coralville, IA, USA). Non-labelled versions of the forward and reverse sequences were also procured for subsequent sequencing library preparation.

For gel electrophoresis, agarose was obtained from Sigma-Aldrich (Dorset, UK). Tris-Acetate-EDTA buffer (TAE ×50) and GelRed nucleic acid gel stain (×10,000 in H_2_O) were obtained from Cambridge Bioscience (Cambridge, UK), and HyperLadder 25 bp and ×6 loading dyes were obtained from Bioline (Toronto, ON, Canada).

For the separation of double-stranded DNA (dsDNA), NeutrAvidin agarose resin was obtained from Thermo Fisher Scientific (Waltham, MA, USA). Corning Costar Spin-X (0.22 μM) and Amicon Ultra 0.5 mL (3 kDa MWCO) filter units were both obtained from Sigma-Aldrich (Dorset, UK). 

The DNA sequencing reagents, such as NEBNext Ultra DNA Library Prep Kit for Illumina, NEBNext Multiplex Oligos for Illumina (all New England Biolabs, Ipswich, MA, USA), Agencourt AMPure XP Beads (Beckman Coulter, Brea, CA, USA) and Qubit^TM^ dsDNA HS Assay Kit, were provided by DNA Analysis at King’s, King’s College London. 

For Circular Dichroism (CD), N1 and N4 aptamers were synthesised with no modification by Sigma-Aldrich (Dorset, UK). CD buffer was prepared through the addition of 5 mM MgCl_2_ to PBS. This was then filtered with a 0.2 μm filter prior to use. A black 10 mm (10 mm × 4 mm) cuvette was used for all measurements. CD was performed using a ChiraScan Plus Circular Dichroism spectrometer (Applied Photophysics, Leatherhead, UK), provided by the Biomolecular Spectroscopy Centre at King’s College London. 

For qPCR assays, N1 and N4 aptamers as well as forward and reverse primers (as used in Cell-SELEX selection) were synthesised with no modification by Sigma-Aldrich (Dorset, UK). The KAPA SYBR^®^ Fast Green qPCR kit was obtained from Scientific Laboratory Supplies (Nottingham, UK). Optical fast plate 96-well qPCR plates were obtained from Scientific Laboratory Supplies (Nottingham, UK) and optical qPCR plate seals were obtained from Starlab (Milton Keynes, UK). Reaction amplifications were carried out using a 7500 Fast Real-Time PCR System (Thermo Fisher Scientific, Waltham, MA, USA) and data was analysed using 7500 Software V2.3 (Applied Biosystems by Life Technologies, Waltham, MA, USA). 

For fluorescent microscopy, N1 and N4 aptamers were synthesised with 5′ 6-FAM groups by Sigma-Aldrich (Dorset, UK). Aptamer binding buffer and aptamer wash buffer were prepared as previously stated. Glass sides (1.0–1.2 mm thick) and glass cover slips (0.13–0.16 mm thick) were obtained from Scientific Laboratory Supplies (Nottingham, UK). Fluoroshield™ was obtained from Sigma-Aldrich (Dorset, UK).

### 3.2. Body Fluid Collection

Human blood and seminal fluid were obtained upon informed consent from healthy donors. All samples were stored at 4 °C until cell isolation and subsequent analysis was conducted. A total of five healthy donors were used, each donating blood twice during the Cell-SELEX rounds (for a total of 10 rounds). All donors were informed, and written consent was obtained. Blood samples were obtained through a finger prick with a safety lancet and collected in an Eppendorf tube containing 3.2% sodium citrate coagulation preservative.

All bodily fluid sample collection and use within this study was conducted in accordance with ethical clearance granted by the King’s College London Biomedical Sciences, Dentistry, Medicine and Natural & Mathematical Sciences Research Ethics Subcommittee (Reference HR-17/18-5057). All research was conducted in accordance with the Human Tissue Act 2004. 

### 3.3. Cell Isolation 

RBCs were isolated from whole blood using a cell washing protocol [26]. Samples were centrifuged at 500× *g* for 10 min before the supernatant, including the buffy coat, was removed and discarded. The RBC pellet was then resuspended in 2 mL of RBC isolation buffer and centrifuged using the same conditions. The supernatant was removed and discarded. A total of 3 washes were performed on the sample prior to the pellet being resuspended in 500 μL of saline. 

Sperm cells were isolated from whole seminal fluid using a density gradient centrifugation protocol [57]. An isotonic gradient was prepared via the addition of 1 mL of ×10 HTF to 9 mL Percoll. Two fractions were then prepared from this solution in x1 HTF: 80% and 40% (*v*/*v*) Percoll suspensions. In a 15 mL tube, 2 mL volume of 80% suspension was added with a 2 mL volume of the 40% suspension layered on top to produce a discontinuous density gradient. A volume of 1 mL seminal fluid was added to the top of the gradient and centrifuged for 20 min at 500× *g*. The supernatant was removed from the pelleted cells, which were then washed twice with 5 mL of ×1 HTF. The final pelleted sperm cells were resuspended in 500 μL SDW.

To determine cell concentration of all cell types, a Countess II automated cell counter (Thermo Fisher Scientific, Waltham, MA, USA) was used. Cells were prepared for counting through the addition of 10 μL of cell suspension to 10 μL of trypan blue solution before being loaded onto a Countess cell counting chamber slide.

### 3.4. Cell-SELEX 

#### 3.4.1. Incubation and Elution

Within the first round of Cell-SELEX, the library was prepared by the addition of 100 μL of 100 μM (10 nmol) DNA library to 270 μL of aptamer binding buffer. The pool was then heated to 95 °C for 5 min and snap cooled on ice until ready to use. For the preparation of RBCs, a solution containing 1 × 10^7^ cells was centrifuged at 500× *g* for 10 min at 4 °C. The supernatant was then removed, and the pellet was resuspended in 330 μL of aptamer binding buffer. The resuspended cells were then added to the 370 μL of snap-cooled ssDNA library. The mixture was incubated for 1 h on a rotary shaker at room temperature. DNA library elution was conducted by centrifuging the mixture at 6700 rpm for 3 min to achieve pelleting of the bound target–aptamer complexes. The unbound sequences within the supernatant were removed, and cells were resuspended and washed three times in 1 mL aptamer wash buffer. Bound ssDNA aptamer sequences were eluted from the cells via the addition of 500 μL of DNAse-free water to the cell pellet. The cells were then heated to 95 °C for 10 min followed by centrifugation at 13,100 rpm for 5 min to pellet the cells. Finally, the supernatant (containing bound sequences) was collected. 

For rounds 2–10, incubation of the library and cells occurred through the addition of 200 μL ssDNA library (diluted to a concentration of 1 μM in aptamer binding buffer) to cells also suspended in aptamer binding buffer. The cell count for rounds 2–6 was reduced in order to increase the selection stringency throughout each round from 1 × 10^7^ down to 1 × 10^6^ cells. The RBC count was then kept constant from round 7 to 10, as negative selection rounds were incorporated. For negative selection from round 7 to 10, a solution of 1 × 10^6^ sperm cells was used per round. Further measures to increase stringency that were used included shortening the incubation period, increasing the number of washes and rotating the five blood donors throughout the 10 completed rounds (Table 1). Elution of bound sequences within rounds 2–10 was achieved through the addition of 600 μL of aptamer binding buffer to the cell pellet prior to heating and centrifugation, as previously mentioned. 

#### 3.4.2. PCR Amplification

During Cell-SELEX, three independent PCR amplifications were performed: preliminary PCR, trial PCR and preparative PCR. Preliminary PCR was only conducted within round 1 of selection on the initially eluted ssDNA pool in order to increase the population of ssDNA within the library and ensure there were multiple copies of each possible bound sequence ahead of the second round of selection. This ensured minimal loss of sequences during subsequent stages of the round. Preliminary PCR was completed by the addition of 500 μL of the eluted sequences to 500 μL of a preliminary PCR mix (Table 4). The mixture was then pipetted into 20 × 50 μL reactions for 9 amplification cycles on a Perkin-Elmer 9700 thermal cycler (Cambridge, UK). Temperature programmes used can be found in Table 4. 

Trial PCR reactions of the eluted aptamer pool for each round of selection were conducted in order to determine the optimum number of amplification cycles needed for the subsequent preparative PCR amplification. A trial PCR reaction mixture was prepared (Table 5) and split into six individual reactions of 45 μL volumes. To each reaction, 5 μL of eluted aptamer pool was added, with the exception of one reaction, to which 5 μL of water was added as a control. Samples were run according to the same temperature programme outlined in Table 4. Tubes were amplified for either 10, 12, 14, 16 or 18 cycles, with the negative control also run for the full 18 cycles. The PCR products were then visualised through the use of a 2.5% agarose gel (made in ×1 TAE with 0.01% GelRed). Samples were run alongside a HyperLadder 25-bp size determination marker and run at 80 V for 100 min in ×1 TAE buffer. 

Finally, to prepare the ssDNA library for the next selection round, the eluted ssDNA pool from each incubation round underwent preparative PCR amplification. Then, 400 μL of each eluted pool was added to the preparative PCR mixture (Table 5) and amplified according to the number of cycles indicated during trial PCR. The reaction mixture was run in 50 μL reactions using the same temperature programme outlined in Table 4. As with trial PCR, gel electrophoresis was used to confirm dsDNA amplification products. 

#### 3.4.3. Strand Separation

Prior to re-incubation of the library with the target cells, the pool underwent strand separation in order to convert the post-amplification dsDNA products into an ssDNA library. The strand separation was conducted with the use of a streptavidin-labelled resin, which when incubated with the dsDNA products, binds to the biotinylated antisense strands. Firstly, 200 μL of Pierce NeutrAvidin agarose resin was added into eight Corning Costar Spin-X filters. The filters were then centrifuged at 2730 rpm for 1 min to remove the storage buffer. The resin was then preconditioned with 500 μL of 200 mM NaOH in order to remove labile avidin molecules for 30 min. The filters were then centrifuged again under the same conditions. All resins were then washed (×5) with 500 μL of ×1 DPBS buffer. To each filter, 500 μL of preparative PCR products was added and left to incubate on a tube rotator for 30 min to allow the dsDNA products to bind to the resin. The resins were centrifuged and washed (×5) in ×1 DPBS with the same conditions, as previously mentioned. Then, 500 μL of 200 mM NaOH was added to each set of beads to separate the fluorescently labelled sense strands from the biotinylated antisense strands. The filters were centrifuged at 2730 rpm for 10 min to collect the supernatant. To purify the ssDNA pool from the NaOH, the collected ssDNA products were placed in x8 Amicon Ultra 0.5 mL (3 kDa MWCO) filters and centrifuged at 14,500 rpm for 30 min. To each filter, 450 μL of DNase-free water was added, centrifuged under the same conditions. Finally, to recover the concentrated solute, the filters were placed upside down in clean microcentrifuge tubes and centrifuged at 3860 rpm for 7 min. The ssDNA pool recovered was lastly quantified using a Cary Eclipse Fluorescence Spectrophotometer (Agilent Technologies, Santa Clara, CA, USA). A sample of the pool was initially diluted 1:50 in DNAse-free water (prepared in triplicate) to a final volume of 60 μL and added to a white 96-well plate (Thermo Fisher Scientific, Waltham, MA, USA). An advanced scan was then conducted using Ex = 495 nm and Em = 520 nm (as per the manufacturer’s recommendations for the use of 6-FAM-labelled ligands). Fluorescent intensities were collected and normalised against a water blank. Using a pre-determined calibration curve, the concentration of ssDNA was calculated. 

### 3.5. Massively Parallel Sequencing

The enriched aptamer pools generated from rounds 6, 8 and 10 of Cell-SELEX underwent MPS in order to elucidate the sequences of the potential binding aptamers. From the three rounds chosen for selection, a PCR amplification reaction was conducted on each pool prior to library preparation for MPS to produce dsDNA products. The same reaction mixture was used as in Table 5 for the preparative PCR, with the exception of the primers, which were substituted for non-labelled primer sets. The same numbers of cycles and temperature programmes were used as in the preparative PCR amplification for that specific round. Amplified products were then quantified using Invitrogen Qubit dsDNA HS quantification kit followed by a dilution of each pool to a concentration of 2 ng/μL. 

The round 6, 8 and 10 libraries were prepared for sequencing with the NEBNext Ultra DNA Library Prep Kit for Illumina. Firstly, 1.4 μL End Prep Enzyme Mix and 2.9 μL End Repair Reaction Buffer (×10) were added to 25 μL of each diluted dsDNA sample and incubated for 30 min at room temperature and then 30 min at 65 °C. The adaptors were then ligated to the ends of the dsDNA products via the addition of 7.5 μL Blunt/TA Ligase Master Mix, 1.25 μL NEBNext adaptor for Illumina and 0.5 μL Ligation Enhancer. The solution was incubated at room temperature for 15 min before 2 μL USER Enzyme was added, followed by another incubation at 37 °C for 15 min. Post-ligation clean-up was carried out using the AMPure XP Beads, with which the sequences were eluted in 10 μL of 10 mM Tris-HCL, (pH 8.5). PCR amplification of the libraries was then conducted, by adding 7.5 μL adaptor-ligated dsDNA to 12.5 μL NEBNext Ultra II Q5 Master Mix, 2.5 μL Universal PCR primer and 2.5 μL Index primer. The PCR mixture was run for 8 cycles in a thermal cycler using the temperature programme outlined in Table 6. 

Finally, clean-up of the library pools was achieved using AMPure XP beads, prior to the elution of samples in ×1 TE buffer. The round 6, 8 and 10 library pools were then loaded into a V2 reagent cartridge and sequenced using the Illumina MiSeq instrument (San Diego, CA, USA). 

Data analysis of the FASTQ files generated by the Illumina MiSeq was conducted using Galaxy, an open-source bioinformatics platform, using a pipeline developed in house that was designed for use with Cell-SELEX library pools following MPS [23,25]. Further analysis was conducted using AptaSUITE, an aptamer-specific bioinformatics software designed for the analysis of MPS aptamer pool files [24]. 

### 3.6. Circular Dichroism (CD)

Both N1 and N4 aptamer sequences were diluted to a stock concentration of 1 μM in CD buffer prior to being loaded to a 10 mm black cuvette (10 mm × 4 mm). CD spectra of the CD buffer were also measured and subtracted from the N1 and N4 sample spectra as background. All measurements were obtained between 400 and 200 nm with 2 mm bandwidth at 23 °C. 

### 3.7. Fluorescent Microscopy

Both N1 and N4 aptamer sequences were synthesised with 5′ 6-FAM groups by Sigma-Aldrich (Dorset, UK) and diluted to a stock concentration of 20 μM in aptamer binding buffer. The solution was heated to 95 °C for 5 min and snap cooled on ice until used. To assess binding to isolated RBC, a stock solution of isolated RBCs was prepared to a concentration of 1 × 10^8^ cells/mL in aptamer binding buffer. A 100 μL volume of each aptamer stock was incubated with 100 μL of RBC suspension and left to incubate for 1 h at 4 °C. After incubation, the solution was centrifuged at 6700 rpm for 3 min to achieve pelleting of the bound target–aptamer complexes. The unbound sequences within the supernatant were removed and the cells were resuspended and washed three times in 1 mL aptamer wash buffer. After the final wash, the cell pellet was resuspended in 100 μL of aptamer binding buffer prior to visualisation. Prior to mounting, 50 μL of Fluoroshield™ was added to the sample in order to enhance stability of the fluorophore and prevent photobleaching during microscopy. 

To assess the binding and stability of N1 and N4 within whole human blood, an unpurified solution of blood was initially diluted 1:10,000 in aptamer binding buffer. A 100 μL volume of each aptamer stock was incubated with 100 μL of whole blood suspension and left to incubate for 15 min at 4 °C. After incubation, the solution was centrifuged at 6700 rpm for 3 min to achieve pelleting of the bound target–aptamer complexes. The unbound sequences, as well as other components of whole blood, within the supernatant were removed and the cells were resuspended and washed three times in 1 mL aptamer wash buffer. After the final wash, the cell pellet was resuspended, as previously detailed.

For both assays, 10 μL of solution was added to a glass microscopy slide and a glass coverslip was used. Slides were visualised under ×40 objective lens using a Nikon Eclipse Ti-2 inverted microscope. Images were captured using a Nikon DS-Qi2 sCMOS camera. ImageJ software (Version 2.14.0/1.54f) was used to analyse the images obtained.

### 3.8. Quantitative Polymerase Chain Reaction (qPCR)

Developed aptamer sequences N1 and N4 were synthesised with no modifications by Sigma-Aldrich (Dorset, UK) and diluted to the following concentrations: 100 nM, 80 nM, 60 nM, 40 nM, 20 nM and 5 nM in aptamer binding buffer. In order to generate a standard curve, aptamer dilutions were prepared in water as a 1:10 serial dilution from 10 nM to 0.1 pM. A positive control of 50 pM was prepared in water to determine the efficiency of the standard curve. All solutions were heated to 95 °C for 5 min and snap cooled on ice until used. A stock solution of isolated RBCs from three healthy donors was prepared to a concentration of 1 × 10^7^ cells/mL in aptamer binding buffer. A volume of 100 μL of stock RBCs was incubated with 100 μL of each aptamer dilution and left to incubate at room temperature for 1 h on a rotary shaker. Reactions were performed in triplicate. Samples were centrifuged at 6700 rpm for 3 min to pellet aptamer–RBC complexes. The cell pellets were washed and re-centrifuged (×3) with 500 μL of aptamer wash buffer. To the final cell pellets, 500 μL of DNase-free water was added before the solution was heated at 95 °C for 10 min. The sample was then vortexed for 10 sec before centrifuged at 13,100 rpm for 5 min. The ensuing supernatant was collected containing bound sequences. Master mix was prepared for all bound aptamer samples (×36), the standard curve samples (×18) and negative and positive controls (×6). Master mix components are summarised in Table 7. 

In an optical fast qPCR plate, a total volume of 20 μL was used: 15 μL of master mix and 5 μL of each sample. The amplification steps can be seen in Table 8 and briefly included an initial enzyme activation step at 95 °C for 3 min, followed by 40 cycles of amplification. All samples were analysed in triplicate, and the data were processed using the 7500 Software V2.3 (Applied Biosystems by Life Technologies, Waltham, MA, USA). The equation corresponding to the standard curve obtained for the pure aptamer was used to calculate the concentration of bound aptamer within the samples. GraphPad Prism (version 9.5.1, GraphPad, Boston, MA, USA) was used to analyse the data obtained. 

## 4. Conclusions

In this study, we successfully selected novel ssDNA aptamers that target human red blood cells, a vital target analyte within forensic analysis and an analyte of interest in a medical setting. Aptamers have previously been developed for a range of surface proteins of the RBC, such as Glycophorin A, the Rhesus D antigenic epitope and A1 antigen, as well as RBCs of medical interest, such as rare circulating foetal RBCs and malaria parasite-infected RBCs [58,59,60,61,62]. However, to the best of our knowledge, this is the first reported use of a modified Cell-SELEX methodology [22] that has used the entire healthy isolated RBC from a pool of donors as the selection target, with negative selection included to increase the stringency of selection. The novel inclusion of negative selection cycles against human sperm cells removes the possibility of cross-reactivity with the other most commonly found biological fluid, which may be a current limitation of existing assays for the detection of blood in a forensic setting. 

The aptamers presented within this work have the potential to be utilised as an analytical probe within a number of fields, notably within forensic science for the detection of human red blood cells. From the pool of 3365 enriched candidates, two aptamers (N1 and N4) were selected for further investigation due to their enrichment profiles. Structural predictions of these candidates demonstrated stable hairpin loop and stem structures, which are similar to one another in their stem and loop confirmation. In addition to the in silico modelling presented within this work, two bioassays were used to characterise binding to the target, in which both aptamers demonstrated their potential for optical labelling as well as dose-dependent binding to RBCs. It is hoped that the aptamers presented within this work will not only have use within forensic science but can be used as a wider analytical tool due to their ability to be incorporated within a wide range of analytical probes or biosensors. 

## Figures and Tables

**Table 1:** Cell-SELEX stringency measures. The stringency measures used within Cell-SELEX in order to increase selection pressure throughout rounds and to ensure that only high-affinity binding ssDNA sequences remain within the binding pool.

Round	Donor	RBC Count	Incubation Time	Washes	Negative Selection
1	1	1 × 10^7^	1 h	3	No
2	2	5 × 10^6^	1 h	3	No
3	3	4 × 10^6^	1 h	3	No
4	4	3 × 10^6^	1 h	3	No
5	5	2 × 10^6^	1 h	3	No
6	1	1 × 10^6^	1 h	3	No
7	2	1 × 10^6^	30 min	3	Yes
8	3	1 × 10^6^	30 min	4	Yes
9	4	1 × 10^6^	30 min	5	Yes
10	5	1 × 10^6^	30 min	6	Yes

**Table 2:** Enriched aptamer candidates. Aptamer candidates generated as a result of 10 rounds of Cell-SELEX that display a level of enrichment within the round 10 binding pool. Variable regions are underlined and highlighted in bold.

	Sequence (5′–3′)
N1	ATCCAGAGTGACGCAGCA**CGGGTTGGGGCTGGTTGTGTGTTGTTTTT** **TTGGCTGTATG**TGGACACGGTGGCTTAGT
N2	ATCCAGAGTGACGCAGCA**TGTGTGAGTTGGATTGAGTGTGGATTGGG** **GGGATGATGGG**TGGACACGGTGGCTTAGT
N3	ATCCAGAGTGACGCAGCA**GGATGGATGTGGTTGGGGATCGGCTGGG** **GGATTTAGTTGG**TGGACACGGTGGCTTAGT
N4	ATCCAGAGTGACGCAGCA**TGCGGGGAGAGGAGTGTGGGATGGGTTT** **GTTTGTTTAGGG**TGGACACGGTGGCTTAGT
N5	ATCCAGAGTGACGCAGCA**ATTTGGAGGCGGGATGATATGCGTAGGGG** **TTTGAGGGGGG**TGGACACGGTGGCTTAGT
N6	ATCCAGAGTGACGCAGCA**GCAGGGTGTGGGGTAAGTGTGTGGGGTG** **TTGTTGTTTGGG**TGGACACGGTGGCTTAGT
N7	ATCCAGAGTGACGCAGCA**CGTGGTGTTTTGGGGGTGTTTGATTGGGT** **TTCTGTTCGGA**TGGACACGGTGGCTTAGT
N8	ATCCAGAGTGACGCAGCA**GGAGAGTTGATTTGGGTGTTGGTGGCTG** **GGATAGTAAGGG**TGGACACGGTGGCTTAGT
N9	ATCCAGAGTGACGCAGCA**TTATGTGGGGGGTGGTGTACTTGTTTGGG** **GATCGGTTGGT**TGGACACGGTGGCTTAGT

**Table 3:** QGRS predictions. QGRS Mapper showed 2 quadruplex-forming G-rich sequences for both N1 and N4. The position on the sequence and length of QGRS is given, as well as the sequence. The guanine groups that form the tetrad are in bold and underlined. The G-score given is an indication of the likelihood of a GQRS to form a stable G-quadruplex [39].

	Position	Length	QGRS	G-Score
N1	21	12	**GG**TT**GGGG**CT**GG**	19
	50	21	**GG**CTGTATGT**GG**ACAC**GG**T**GG**	14
N4	22	22	**GG**GGAGA**GG**AGTGT**GG**GATG**GG**	20
	57	14	**GG**T**GG**ACAC**GG**T**GG**	18

**Table 4:** Thermal cycling conditions. Preliminary, trial and preparative PCR amplification conditions used within Cell-SELEX. * Cycling stages.

Amplification Step	Temperature (°C)	Time (s)
Denature	95.0	120
Denature *	95.0	30
Annealing *	56.3	30
Extension *	72.0	30
Extension	92.0	180
Hold	4.0	∞

**Table 5:** PCR reaction mixtures for SELEX.

Reagents	Preliminary PCR (μL)	Trial PCR (μL)	Preparative PCR (μL)
X10 PCR Buffer	100.00	35.00	400.00
dNTP Mixture (10 mM)	20.00	7.00	80.00
MgCl_2_ (50 mM)	40.00	14.00	160.00
Forward Primer (100 μM)	5.00	1.75	20.00
Reverse Primer (100 μM)	5.00	1.75	20.00
DNAse-Free Water	327.00	254.45	2908.00
Platinum Taq Polymerase	3.00	1.05	12.00
Eluted ssDNA Pool	500.00	-	400.00

**Table 6:** Library preparation thermal cycling. Thermal cycling conditions for MPS library preparation. * Cycling stages.

Amplification Step	Temperature (°C)	Time (s)
Denature	98.0	30
Denature *	98.0	10
Annealing *	65.0	75
Extension *	65.0	300
Hold	4.0	∞

**Table 7:** PCR reaction mixtures for qPCR.

Reagents	Per Sample (μL)	Total (μL)
PCR Grade Water	4.0	240.0
KAPA SYBR^®^ Fast Green Master Mix (2×) Universal	10.0	600.0
Forward Primer (10 μM)	0.4	24.0
Reverse Primer (10 μM)	0.4	24.0
50× Low ROX	0.2	12.0
Template ssDNA	5.0	-

**Table 8:** qPCR thermal cycling. Thermal cycling conditions qPCR. * Cycling stages.

Amplification Step	Temperature (°C)	Time (s)
Enzyme Activation	95.0	180
Denature *	95.0	10
Annealing and Extension *	65.0	30

## Data Availability

Data is contained within the article.
